# Drs. Frank Abbott and L. G. Ingersoll

**Published:** 1898-01

**Authors:** Truman W. Brophy, J. H. Wooley, C. S. Case

**Affiliations:** Committee.; Committee.; Committee.


### THE LATE DRS. FRANK ABBOTT AND L. G. INGERSOLL.

The following resolutions were passed at a meeting of the Chicago Dental Society, October 5, 1897.

WHEREAS, again we are called upon to record the name of a distinguished American dentist among those whose life work is finished and whose achievements form a conspicuous part of the history of dentistry the last quarter of a century.

The life of Dr. Frank Abbott was one of great worth to dentistry in America. During a period of about thirty years he was at the head of the New York College of Dentistry, and with this institution—one of the foremost in our country—his name will ever be inseparable.

Dr. Abbott was honored by election to the presidency of the American Dental Association, the Association of Dental College Faculties, and other bodies with which he was connected, and was recognized as one of the leading men in the profession.

*Resolved*, That in the death of Dr. Frank Abbott the dental profession has lost a member whose affiability won him our friendship, and whose earnest efforts in advancing dentistry among practitioners and as an educator placed him among the foremost men in the profession; therefore, be it

*Resolved*, That the Chicago Dental Society deeply regrets the untimely death of Dr. Abbott, and conveys herewith its sympathy to his family in their bereavement.

*Resolved*, That a copy of these resolutions be spread upon our records and a copy be sent to the dental journals and to his family.

WHEREAS, The change so rapidly taking place among the pioneers of our profession has been conspicuously marked in the death of Dr. L. G. Ingersoll, of Keokuk, Iowa.

Dr. Ingersoll was a leader in the profession and contributed largely to its literature. His standing as a man of broad education gave him a prominent place among educators and commanded the attention of all audiences which he addressed. Dr. Ingersoll was not only a scholar, but he was an earnest worker and an eloquest speaker.

*Resolved*, In the death of Dr. Ingersoll the profession has lost another distinguished member, whose life work was in the interest of a higher educational standard.

*Resolved*, That a copy of these resolutions be placed on the records of this Society and published in the dental journals, and that a copy be forwarded to his family.

TRUMAN W. BROPHY,
J. H. WOOLEY,
C. S. CASE,
*Committee.*

---

